# The structure and evaluation of educational research skills and accomplishments among rural teachers: Data from China

**DOI:** 10.3389/fpsyg.2023.955921

**Published:** 2023-03-02

**Authors:** Luo Feng, Yao Yuxiang, Qiu Lihua

**Affiliations:** ^1^Educational Scientific Institutes, Hunan Normal University, Changsha, China; ^2^Primary Education Institutes, Changsha Normal University, Changsha, China

**Keywords:** educational research, rural teachers, educational research, teacher evaluation, teacher development, regional norm

## Abstract

The practice of educational research by rural teachers is highly valued and very important for their professional development and for the revitalization of rural education. This study explored the components of educational research activities among rural teachers (Study 1). Based on the results, a regional norm for Hunan province was formulated, and discriminant criteria were developed for the evaluation of educational research skills and accomplishments among rural teachers (Study 2). In Study 1, data from 892 Chinese rural teachers working at compulsory education schools in Hunan Province (a representative province in central China), divided into two samples, were found to support the constructs included in the measurement instrument. Exploratory and confirmatory factor analyses of the 33 items of the Rural Teachers’ Educational Research Self-rating Scale identified a first-order model with three factors: educational research on basic educational activities (BEA), educational research involving the creation of an educational community (CEC), and educational research involving the refinement and popularization of educational theory (RPE). Based on the results of Study 1, in Study 2, a set of norms for educational research skills and accomplishments among rural teachers was formulated based on the data from Hunan Province. This norm can serve as a reference standard for the evaluation of rural teachers’ educational research skills and accomplishments. The components of rural teachers’ educational research activities are discussed, and suggestions for the formulation of education policies are provided.

## 1. Introduction

As early as 1926, Buckingham (1926) began to discuss the topic of “The Teacher as Research Worker.” “Educational research” refers to the activities that teachers engage in and the systematic methods that they employ in order to explore educational phenomena or problems (Liu, 2015; Zheng, 2019). Teachers’ engagement in educational research has several specific and valuable effects, such as enriching human spiritual culture, stimulating organizational vitality, forming organizational cohesion, and continuously improving people’s quality of life (Yang, 2005). In recent years, ways to improve the level of engagement with educational research among teachers has become the focus of China’s basic education community (Meng and Xing, 2001). In contrast with the earlier concept of educational research in China, in which it was regarded as a task for college teachers, educational research among compulsory education^1^ teachers has gradually received more attention. Most scholars assume that the key to improving levels of educational research among compulsory education schoolteachers is their literacy in the domain of educational research. Relevant issues include the components of educational research literacy (Soto Gómez et al., 2019), the current reality of educational research literacy (Jia and Wang, 2009), and the strategies employed to improve the quality of educational research literacy (Dong, 2008). However, the structure of educational research activities has not been sufficiently explored. More importantly, in China, the number of compulsory education students in rural areas accounts for 66% of all students in compulsory education; however, the academic community knows very little about educational research among rural teachers.

### 1.1. Structural components of educational research

To better understand and evaluate teachers’ skills and accomplishments in the domain of educational research, the structural components of educational research must be analyzed. Several structural schemas have been previously proposed. (1) Based on the purpose and theoretical level of educational research, Jia et al. (2011) divided the concept into basic research, applied research, and developmental research. Similarly, Tang and Hu (2015) divided educational research into basic research, applied research, and comprehensive research. (2) Based on the educational research paradigm, Cai (2011) and Wei et al. (2012) classified educational research activities as either qualitative research or quantitative research. While these divisions are directly derived from the structure of “research,” they lack the specific pertinence to “education.” In addition, “action research” is a form of research that differs from traditional academic educational research (Liu, 2001), in that it is characterized as “research for action, research by actors, research in action” (Chen, 2001) and places the “focus on actors’ self-reflection” (Kemmis et al., 1982). This research paradigm has strong applicability for elementary education teachers, which was verified in a pre-investigation that formed part of this study.

It is educationally appropriate to explore the structural components of educational research carried out by compulsory education teachers from the perspective of the action research paradigm. Research targeting individual educational practice (including teaching and management activities) is the most basic and common form of educational research (Kemmis et al., 1982; Cheng, 2011; Nami and Matin, 2017). However, it has been suggested that in addition to educational research targeting teachers’ individual educational practices, educational research with collective significance and a community nature, in the form of collaboration and dialog, is also important (Curry et al., 2018). This kind of research best demonstrates the value of criticizing educational science (Carr and Kemmis, 2003), which creates meaning for teaching practice through collaborative reflection. In addition, the processes of compilation and promotion of the educational experience can also be considered as educational research for elementary education teachers. This view emphasizes that teachers can refine theories that can be popularized based on practical experience (Elliot, 1991; Tang, 2011). In summary, although the above-mentioned objects of educational research have been addressed before (Somekh and Zeichner, 2009; Song, 2021), integrated empirical research on the structure of educational research based on the object of research is still underdeveloped. As a result, existing research findings do not provide a sufficient basis for understanding the structural characteristics of teachers’ educational research, evaluating teachers’ educational research accomplishments, and guiding teachers to carry out educational research.

### 1.2. Educational research among rural teachers

Most of the abovementioned studies focus on urban teachers; with regard to educational research among rural teachers, the situation will be very different. Generally, rural teachers seem not to be very interested in educational research, especially in China. The main reasons are summarized below.

The first of these reasons relate to the external environment. First, the workload of rural teachers is heavy. Zhao (2019) surveyed rural teachers in 23 provinces of China and found that these teachers generally suffer a heavy workload despite their low salary. Therefore, they have no time or energy left to accept teaching and research support and often face serious levels of emotional exhaustion. Second, funding for rural education is insufficient. At present, China’s compulsory education is funded *via* a “county-based” mode of investment in education, which means that investment in funding of education depends on the income of the county where the school is located (Qu, 2017). The funds provided to rural schools are therefore far lower than those of urban schools, which makes them inadequate to guarantee that rural teachers will be able to carry out educational research or encourage them to do so.

Other reasons involve factors relating to the teachers themselves. First, rural teachers often lack the motivation to conduct educational research (Swanson, 2011; Richardson, 2017). Second, the ability of rural teachers to conduct scientific educational research involving papers and projects is relatively weak (Lu et al., 2016). Third, in China, most rural teachers have not yet fully realized the value of educational research in resolving their own difficulties (Zhang, 2020).

Given this realistic assessment of the situation, the structure and quality of rural teachers’ educational research in China may be distinct from those of other teachers. Important questions are: what do the structural components of educational research carried out by rural teachers in China look like, and how can the educational research skills and accomplishments of rural teachers be measured? Addressing these questions will help to establish a more realistic evaluation mechanism and guidance strategy for educational research among such teachers. This was the purpose of the present study.

### 1.3. Research objectives

In order to explore the structural components of educational research among rural teachers in the context of China and to assess their current skills and accomplishments in this domain, this study focused on Hunan Province, a large province in central China with a representative educational structure. This study consists of two main parts. First, semi-structured interviews were conducted with rural teachers to collect information on their educational research activities; subsequently, the structure of educational research was explored and verified, and a scale for measuring educational research among rural teachers was developed. Second, on the basis of rural teachers’ current skills and accomplishments in educational research, a set of norms and discriminant criteria were constructed for the evaluation of rural teachers’ educational research skills and accomplishments, and a practical evaluation standard for educational research among rural teachers was established.

## 2. Study 1: Structure of educational research among rural teachers

### 2.1. Preliminary investigation

The purpose of the preliminary investigation was to collect information on educational research activities among rural teachers in China in preparation for analysis of the structural components of rural teachers’ educational research activities and the construction of a scale to measure rural teachers’ skills and accomplishments in educational research. Through analysis of the literature and semi-structured interviews with target groups, an initial questionnaire for the evaluation of rural teachers’ educational research skills and accomplishments was designed. Two sample interview questions are: “Which methods do you use to conduct educational research?” and “What has your educational research achieved?” The interview content was then transcribed. Analysis of these interview results showed that interviewees generally talked about the methods they used in specific educational situations and the results they obtained. Therefore, educational research activities were described according to the sentence pattern: “method + object + outcome.” The results showed that the methods and outcomes of educational research are highly consistent. In particular, interviewees often identified the outcomes of conducting academic research as “publishing a paper” and “applying for funding.” However, when the research method reported by the respondent fell under the action research paradigm, their description of the outcomes was transformed into examples of the practical effects of the research, such as effects on the academic performance of students and the classroom atmosphere.

Following this analysis, 45 educational research activities were identified according to the object of the research and its outcomes. These activities were organized into items (including several items referring only to the overall outcome of the research, such as “My students seem to like me”); these items were to be rated on a 5-point Likert scale, with 1 indicating non-conformance and 5 indicating conformance, the response options being arranged in order from 1 to 5. Subsequently, 10 rural teachers were asked to assess the comprehensibility, plausibility, and completeness of the items. Finally, an initial version of the Educational Research Self-rating Scale for Rural Teachers (ERSS-RT), a questionnaire containing 45 items, was formulated.

Initially, 104 of these questionnaires were randomly distributed among rural teachers as a preliminary survey. Responses to all 104 questionnaires were collected, 88 of which were valid (on the basis of responses to lie detection questions and ambivalent demographic data). Preliminary factor analysis showed that nine items under the category of academic educational research were more weakly correlated (<0.3) with other items and had lower loadings (<0.5) onto their respective factors. This indicated that these items were not suitable to be pooled with the other items, suggesting that academic research activities form a distinct component of educational research among rural teachers, and this component is not suitable for inclusion in a more general discussion. Therefore, 36 of the 45 items were retained for the second version of the scale.

To establish criteria for the evaluation of the validity of such a scale, experts have suggested that the general standards and regulations of professional title evaluation documents can be used as calibration items (Hunan Education Department, 2017). These documents use the honors awarded to teachers in recognition of their practical activities as criteria for the evaluation of teachers’ professional titles. After discussing the rationale and universality of these standards with 10 rural teachers, 11 general criteria were selected (these included items relating to the professional standards applied by most provinces, cities, and prefectures). These criteria partly reflected rural teachers’ educational research achievements. Subsequently, the wording of the items and the corresponding response options was discussed with 10 rural teachers: for example, for the item “In the teaching competitions I have participated in, the highest-level award I have won is:,” the response options included “no participation or no award,” “school level,” “district or town level,” “county or city level,” and “provincial level or above.” Finally, responses were extracted and the first version of the 11-item Educational Research Achievements Questionnaire for Rural Teachers (ERAQ-RT) was compiled.

Prior to the questionnaire-based investigation, several demographic questions, two lie-detection questions, the second version of the ERSS-RT, and the first version of the ERAQ-RT were sent to rural teachers who had not participated in the first preliminary survey or the above-described interviews. A total of 100 responses to the questionnaire were collected, 89 of which provided valid data for the calculation of descriptive statistics and for exploratory factor analysis. The questionnaire was not further revised because the factor loading matrix coefficients of all items were > 0.5, meeting the statistical requirements for factor analysis.

### 2.2. Formal investigation

#### 2.2.1. Participants and sampling

A total of 850 questionnaires were randomly distributed to teachers; these consisted of demographic questions (7 items), the second version of the ERSS-RT (36 items), the first version of the ERAQ-RT (11 items), and 2 lie detection questions. The teachers who received them worked in four types of schools: village primary schools, central town primary schools, town junior middle schools, and village junior middle schools. A total of 803 valid responses to the questionnaire were collected (for an effective response rate of 94.47%). All data were analyzed using SPSS (version 23) and Mplus (version 8.3). All participants were from Hunan Province, and none of them had previously participated in the preliminary interviews or investigation. The 803 valid questionnaire responses were pooled with the above-mentioned 89 valid responses, producing a total of 892 responses for statistical analysis. Among these, 228 of the respondents (25.6%) were men and 664 (74.4%) were women. The proportions of respondents with no title, a junior title, a mid-level title, and a senior title were 31.3%, 29.7%, 31.2%, and 7.8%, respectively. The distribution of teacher seniority was as follows: 383 respondents (42.9%) had up to 3 years of teaching experience, 126 (14.1%) had 3 to 5 years of experience, 114 (12.8%) had 6 to 10 years, 40 (4.5%) had 11 to 15 years, 50 (5.6%) had 16 to 20 years, and 179 (20.1%) had more than 20 years of experience.

#### 2.2.2. Item analysis

Two forms of analysis were conducted: a qualitative analysis, in which content validity was assessed, and a quantitative analysis, in which item difficulty and item discrimination were considered. Pearson correlation coefficients were calculated as a measure of the correlation between each item and the total score; these results indicated whether each item represented a valid component of “rural teachers’ educational research activities.” Item discrimination was analyzed primarily *via* an independent-samples *t*-test, where the resulting t value was the critical ratio. All *p* values of both analyses were highly significant (*p* < 0.001); the correlation coefficients fell within the range of 0.68 to 0.87, and the t values ranged from −40.85 to −25.87. These results showed that these items were valid components of rural teachers’ educational research activities and each individual item had a certain discriminative power.

#### 2.2.3. Exploratory factor analysis

The full set of valid questionnaire responses was equally and randomly divided into two samples. Sample 1 (*N* = 446) was used for exploratory factor analysis, and Sample 2 (*N* = 446) was used for confirmatory factor analysis. An independent-samples *t*-test showed that there was no significant difference between Sample 1 and Sample 2 in terms of total ERSS-RT scores (*t* = −0.54, *p* = 0.59). Tests of the suitability of Sample 1 for exploratory factor analysis showed that the KMO value was 0.98, and χ2 reached the threshold for significance (*p* < 0.001) in Bartlett’s test of sphericity, which indicated that the data were suitable for exploratory factor analysis. Considering that the pairwise correlations between items reached significance (*r* = 0.33–0.87), principal axis factoring with oblique rotation was employed (Tabachnick et al., 2007).

Through exploratory factor analysis, three factors with feature roots greater than 1 were extracted, and the total cumulative proportion of the variance explained was 75.67%. The factor loading of item 25 was the lowest (<0.4), and items 12 and 24 were cross-loaded onto two factors; all three of these items were removed. Exploratory factor analysis was repeated for the remaining 33 items, using the same steps as described above. The results showed that there was almost no change in the KMO value or the outcome of Bartlett’s test of sphericity, and three factors accounted for 76.4% of the total variance. This indicated that these three factors had the greatest explanatory power in terms of the structure of educational research among rural teachers. The communality of items exceeded 50%, and all coefficients for each item in the factor loading matrix exceeded 0.55 (range: 0.59–0.97). Table 1 shows the factor loadings for each item.

**Table 1:** Exploratory factor loadings for each item in Sample 1 (*N* = 446).

Item	Factor 1 (BEA)	Factor 2 (CEC)	Factor 3 (RPE)
1. I continue to study teaching methods, so that teachers and students can interact harmoniously in the classroom.	0.722		
2. I actively study and use new teaching software (technology or platforms) to achieve better teaching.	0.728		
3. When teaching, I am good at planning and diligent in reflection.	0.775		
4. I have a comprehensive understanding of students and teach students according to their aptitudes.	0.653		
5. I lead students to achieve better academic results.	0.774		
6. I have mastered classroom dynamics and improved classroom management.	0.854		
7. I patiently think about the reasons for students’ lack of discipline and guide students who are not disciplined.	0.854		
8. In classroom management, I constantly think about management methods, and predict and solve management problems.	0.812		
9. I carefully explore methods of educating people so that they develop a good character.	0.768		
10. I pay timely attention to the psychological dynamics of students and help them grow up healthily.	0.816		
11. I have explored effective ways to communicate with students’ parents.	0.589		
13. Through observation and exploration, I have gained a deeper understanding of local rural culture.		0.659	
14. I weave rural elements into the classroom.		0.819	
15. I organize class activities based on rural culture.		0.832	
16. I am concerned with and think over certain problems in the development of rural education and have independent opinions.		0.836	
17. Under my guidance, my students come to better understand and love the countryside.		0.808	
18. I have explored ways to help my colleagues improve their professional level.		0.728	
19. I actively provide suggestions for teaching, research offices, and schools.		0.701	
20. I constantly adjust, realize, and surpass the original role orientation of teachers in practice.		0.685	
21. I have witnessed problems in school management and actively seek strategies for improvement.		0.773	
22. I actively participate in the future planning of the school.		0.611	
23. I use rural resources to make and develop teaching tools to promote teaching.		0.591	
26. I have developed and implemented a school-based curriculum.			0.677
27. My educational papers, research reports, and experience introduction have been promoted.			0.737
28. I have been invited to demonstrate my teaching ability in various open classes and demonstration classes.			0.849
29. I am often invited to share my experience in moral education and management activities.			0.847
30. I have performed well in refresher courses or heterogeneous forms for the same subject.			0.836
31. My teaching plans and course materials are often used as a reference by colleagues.			0.811
32. I share educational essays through various channels (blogs, WeChat circles, etc.).			0.700
33. My students seem to like me.	0.926		
34. My colleagues seem to recognize my professional ability.	0.966		
35. My students’ parents have a high overall evaluation of me.	0.850		
36. I think I am a rural teacher who continues to explore and grow.	0.793		

BEA, educational research on basic educational activities; CEC, educational research involving the creation of an educational community; RPE, educational research involving the refinement and popularization of educational theory.

As shown in Table 1, items 1–11 and items 33–36 loaded onto factor 1. These items mainly reflect educational research activities and their effectiveness in terms of daily teaching, student management, and home–school cooperation, as well as other educational situations. Items 13–23 loaded onto factor 2. These items mainly reflect educational research activities and their effectiveness in terms of helping with the development of colleagues, teaching and research offices, schools, villages, and other educational communities. Finally, items 26–32 loaded onto factor 3. These items mainly reflect educational research activities and their effectiveness in the educational context of the further refinement and popularization of the respondent’s own experience.

According to the factor loading values and existing theoretical research, the three factors were defined as follows. Factor 1: educational research on basic educational activities (BEA), which includes carrying out daily teaching work, management work, and home–school cooperation. Factor 2: educational research involving the creation of an educational community (CEC), which involves understanding rural culture and carrying out various educational activities based on its characteristics. Factor 3: educational research involving the refinement and popularization of educational theory (RPE), which involves developing curricula, writing educational papers, and designing teaching plans. The teaching and research level of rural teachers is reflected in various kinds of knowledge products that they create in their daily educational activities.

#### 2.2.4. Confirmatory factor analysis

Mplus 8.3 was used to analyze the Sample 2 data to test the rationale underlying the structural components of educational research carried out by rural teachers and the structural validity of the scale. The structural fit indices for the three-factor model each reached an acceptable level (χ2/df = 3.8, *p* < 0.01; CFI = 0.92; TLI = 0.91; RMSEA < 0.08). Exploratory factor analysis showed that the proposed structure was an ideal model of educational research activities among rural teachers. In addition, the second-order factor model was equivalent to the first-order three-factor model because of their equal degrees of freedom. Psychological plausibility and a good model fit led to the selection of the first-order model.

**Figure 1:**
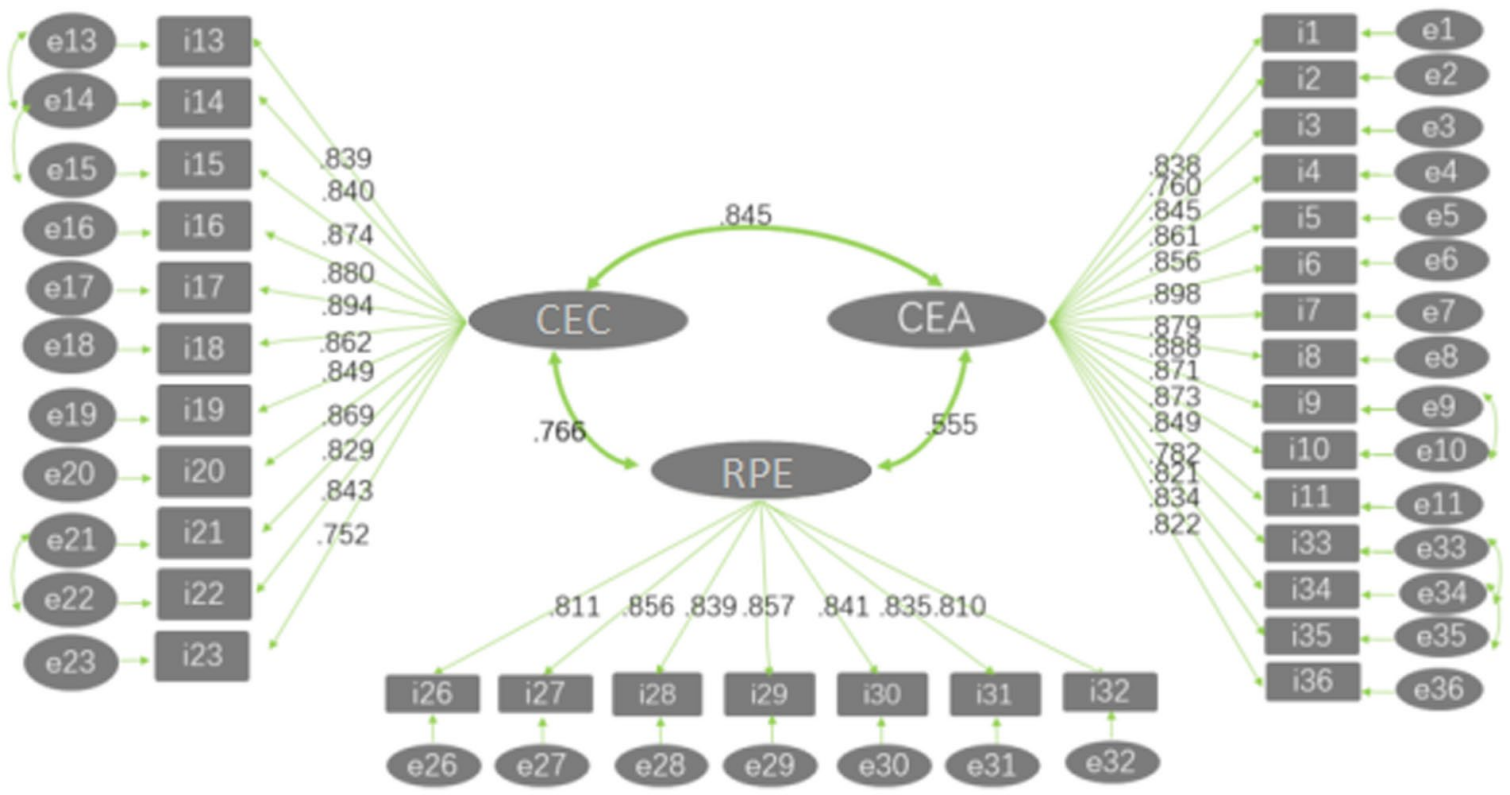
Structure of the scale.

The underlying structure of questionnaire items according to this model is shown in Figure 1.

With regard to measurement errors, a possible cause may be the overlap in content and expression between items 9 and 10, between items 13 to 15, between items 21 and 22, and between items 33 to 35. In addition, these items were presented consecutively in each case, meaning that respondents could easily have become stuck in a certain mindset when filling in these sections of the questionnaire. Considering that each of these item has its own emphasis, all of them were retained. However, in subsequent use of the scale, it is important to reorder the items to split up those that are closely related.

#### 2.2.5. Further examination of the structural components of educational research

Given the high importance of validity in examining measurement models, the reliability and validity of the resulting factors were assessed following the establishment of the measurement model. Prior to this assessment, in order to test whether significant common method bias was present, Harman’s single-factor test was conducted under principal components analysis and confirmatory factor analysis. The cumulative variance contribution rate of the first factor was 49.61%, which is below the standard threshold of 50%, as proposed by Hair et al. (1998). Fit indices showed a relatively poor model fit for the one-factor model, χ2/df = 14.04, CFI = 0.65, TLI = 0.64, RMSEA = 0.13 (Wen et al., 2004). In conclusion, the dataset was not strongly affected by common method bias.

A reliability assessment requires estimates of composite reliability (CR) and average variance extracted (AVE) for each variable. The values of CR and AVE should be ≥0.70 and ≥ 0.50, respectively. Thus, in a measurement model, a construct is considered reliable if its loading value is at least 0.50 (Bagozzi and Yi, 2012). As shown in Table 2, CR scores for each construct ranged from 0.94 to 0.97 and AVE scores ranged from 0.70 to 0.72, both exceeding the suggested cut-off values of 0.70 and 0.50, respectively (Table 2).

**Table 2:** Reliability and validity tests.

Construct	Cronbach’s α	Composite reliability	Average variance extracted
BEA	0.972	0.974	0.716
CEC	0.967	0.966	0.721
RPE	0.952	0.942	0.698

Discriminant validity represents the extent to which a particular construct in the model is uniquely different from other constructs (Hair et al., 2014). The discriminant validity of each construct was tested by comparing the square root of the AVE with the correlations among latent variables. The results of a comparison between all correlations with the square root of the AVE (as shown in Table 3) indicated that discriminant validity was established, as the values of the square root of the AVE (diagonal elements) exceeded those of the construct correlations (off-diagonal elements; Fornell and Larcker, 1981). These findings show that the measurement model was both valid and reliable.

**Table 3:** Test of the discriminant validity of potential variables.

Potential variable	Mean	SD	BEA	CEC	RPE
BEA	4.130^a^	0.765	**0.846** ^b^		
CEC	3.775	0.919	0.821	**0.849**	
RPE	3.142	1.188	0.546	0.740	**0.836**

a The scale was reverse-coded (1 = very inconsistent; 5 = very consistent).

b The bold diagonal elements are the square roots of each AVE; construct correlations are shown off-diagonal.

#### 2.2.6. Analysis of the reliability and validity of the scale

Previously presented results have highlighted the excellent reliability and validity of the scale: Cronbach’s α values were above 0.95, as shown in Table 2, indicating that the reliability of the scale in terms of internal consistency was high; the scale development process indicated that the scale had reliable content validity; and the data presented in Sections 2.2.2–2.2.5 show that the scale had good structural validity.

To further verify the reliability and effectiveness of the ERSS-RT scale developed in this study, ERAQ-RT scores were used to calibrate the ERSS-RT and the relationship between these scales was examined. Although the ERAQ-RT is an ordinal scale, scores on this scale were statistically analyzed by treating ordered categorical variables as continuous variables (Johnson and Creech, 1983). Factor analysis was conducted on data from Sample 1 and Sample 2, which did not differ in terms of total scores (t = −0.47, *p* = 0.64). The results showed that the items of the ERAQ-RT can be treated as falling into a single factor (KMO = 0.92; Bartlett’s test of sphericity coefficient = 0.000; total variance contribution rate = 46.97%). The results of confirmatory factor analysis (χ^2^/df = 3.1, *p* < 0.01; CFI = 0.96; TLI = 0.95; RMSEA = 0.07) indicated a good model fit. However, the communality of item 10 (relating to publishing papers, monographs, etc.) was below 0.3. After this item was removed, the results of exploratory factor analysis and confirmatory factor analysis produced higher fit indices (KMO = 0.93; total contribution rate of variance = 48.9%; χ2/df = 2.96, *p* < 0.01; CFI = 0.97; TLI = 0.96; RMSEA < 0.07); the selected internal consistency coefficient of calibration items was 0.90; and the reliability was good. These findings upon removal of item 10 also indicate that abstract and theoretical achievements (such as papers and monographs) are not the main form of educational research achievements among rural teachers. Analysis of the correlation between the ERSS-RT and the ERAQ-RT showed that both factor scores and total scores (hereafter referred to as ERSS) on the ERSS-RT were positively correlated with total ERAQ-RT scores (r_BEA_ = 0.262; r_CEC_ = 0.268; r_RPE_ = 0.224; r_ERSS_ = 0.206; all *p*-values < 0.001), indicating that the calibration validity was good.

Overall, the ERSS-RT scale developed based on the structural components of rural teachers’ educational research was found to have good reliability and validity, and it can be used as a tool to evaluate rural teachers’ educational research skills and accomplishments.

## 3. Study 2: Formulation of criteria for evaluation of rural teachers’ educational research skills and accomplishments in the Chinese context

In Study 1, the theory that action research is the more suitable form of educational research for rural teachers was verified, and it was found that rural teachers’ educational research involves three types of activity with different objects of research. In Study 2, norms and criteria were constructed on the basis of the levels achieved by rural teachers in these three types of research. It is worth pointing out that the relationships between the seven demographic variables mentioned above and the rural teachers’ educational research skills and accomplishments were not specifically reported, because (with the exception of length of teaching experience), none of the other variables were found to have a stable causal relationship with educational research scores. However, ERSS-RT total scores and individual factor scores of the scale were confirmed to be affected by years of teaching experience (F_BEA_ = 35.86, F_CEC_ = 19.60, F_RPE_ = 4.21, F_ERSS_ = 23.32; all *p*-values < 0.001). Therefore, sub-group norms were established for three groups according to years of experience (early-career teachers: less than 3 years of experience; mid-career teachers: 4–10 years of experience; senior teachers: more than 10 years of experience). Norms and criteria were formulated separately for each stage of teaching experience.

### 3.1. Establishing a regional norm for educational research accomplishments among rural teachers in China

A total of 892 valid questionnaire responses were obtained from teachers working in China’s Hunan province, which is located in central China, a large province in terms of education provision, and a source of representative educational data. This sample can be considered to suitably reflect the level of rural teachers’ educational research in Hunan Province and the average level across China. On this basis, the regional norm for each stage of teaching experience in Hunan Province was established; the results are shown in Table 4.

**Table 4:** Regional norms for educational research skills and accomplishments among rural teachers in Hunan Province.

Scale/subscale (one-way analysis of variance)	Teaching experience	Number of respondents	Average score	Standard deviation
BEA (*F* = 35.86^***^)	Early-career	383	58.49	11.331
	Mid-career	240	63.28	11.157
	Senior	269	65.68	10.529
CEC (*F* = 19.60^***^)	Early-career	383	39.38	9.796
	Mid-career	240	41.85	10.276
	Senior	269	44.29	9.730
RPE (*F* = 4.21^*^)	Early-career	383	21.13	8.117
	Mid-career	240	22.22	8.585
	Senior	269	23.01	8.256
ERSS (*F* = 23.32^***^)	Early-career	383	119.01	26.066
	Mid-career	240	127.35	27.350
	Senior	269	132.99	25.413

* *p* < 0.05;

** *p* < 0.01;

*** *p* < 0.001.

As this research is pioneering, there is currently no norm to which rural teachers can refer directly. The primary criteria for evaluation of rural teachers’ educational research skills and accomplishments were their average scores on the BEA, CEC, and RPE dimensions and overall ERSS scores. According to the results of the data analysis, rural teachers across all three groups scored on average 3.9–4.4 points on the 15 BEA items, 3.6–4.0 points on the 11 CEC items, and 3.0–3.3 points on the 7 RPE items. Overall, rural teachers across all three groups scored an average of 3.6–4.0 points per item on the full 33-item ERSS. Based on the response options provided for each of the items, a score of 3 represents general conformance, 4 represents comparative conformance, and 5 represents complete conformance. The average score of rural teachers was more than 3 points. Therefore, it can be inferred that educational research skills and accomplishments among rural teachers in China are good, and their level increases with increasing teaching experience.

### 3.2. Classification of rural teachers by educational research skills and accomplishments and discrimination of their level of accomplishment

To clarify the role of the norms developed above in evaluation of rural teachers, the topic of classification and discrimination of educational research level was explored to help teachers judge their relative educational research level more readily. Standardized scores on each of the three factors, plus total scores, were taken as clustering variables for a k-means cluster analysis. The sample of rural teachers was divided into three clusters on the basis of educational research level; the distance between the cluster centers was found to be extremely significant for scores on all three factors and total scores (*p* < 0.000). This indicated that it was feasible to divide rural teachers into three classes on the basis of educational research level. Class I respondents had low scores on each of the three factors and thus belonged to a low-level group; Class II respondents had mid-level scores and thus belonged to a mid-level group; and Class III respondents had high scores and belonged to a high-level group. The proportions of respondents within each cluster falling into each group in terms of length of experience are shown in Table 5.

**Table 5:** Cluster-based classification of rural teachers on the basis of educational research skills and accomplishments, and the distribution across teaching experience groups within each cluster.

Classification	Center value				Teaching experience distribution by cluster		
	Z(BEA)	Z(CEC)	Z(RPE)	Z(ERSS)	Early-career	Mid-career	Senior
Class I	−1.196	−1.202	−0.910	−1.244	57.87%	23.62%	18.50%
Class II	0.137	0.059	−0.121	0.043	41.84%	26.53%	31.63%
Class III	1.017	1.147	1.133	1.216	29.27%	30.89%	39.84%

The results presented in Table 5 show that more than half of Class I respondents were in the initial stages of their teaching career, and nearly 40% of Class III respondents were in the senior stage. This indicates that the teaching experience affects teachers’ overall level of educational research accomplishments.

Taking scores on each factor and total educational research achievement scores as discriminant variables, discriminant functions were constructed for each of the teaching experience groups and for all groups to enable interested individuals or organizations to quickly judge a teacher’s level of educational research achievement. In the process of constructing these functions based on individual factor scores, two canonical discriminant functions were found to effectively classify the respondents (*p* = 0.000), but the index of the first function was much larger than that of the second (the first function explained 99%, 98.7%, and 98.9% of the variance among the early-career, mid-career, and senior teachers, respectively). Therefore, only the first function was analyzed. Table 6 provides the specific functions generated.

**Table 6:** Discriminant functions for classification of rural teachers based on educational research skills and accomplishments.

	Fisher function based on standardized factor scores	Fisher function based on standardized total scores
Early-career (98.2%)	*F*(1) = −4.25 * Z(BEA) − 2.72 * Z(CEC) − 2.34 * Z(RPE) − 6.28	F(1) = −8.31 * Z(ERSS) − 6.17
	*F*(2) = −0.16 * Z(BEA) + 0.30 * Z(CEC) − 0.22*Z(RPE) − 1.11	F(2) = −0.03 * Z(ERSS) − 1.10
	*F*(3) = 3.64 * Z(BEA) + 2.40 * Z(CEC) + 3.35 * Z(RPE) − 6.29	F(3) = 8.31 * Z(ERSS) − 6.17
(99%)		
Mid-career (98.3%)	F(1) = −2.47 * Z(BEA) − 4.99 * Z(CEC)-1.87 * Z(RPE) − 6.7	*F*(1) = −8.24 * Z(ERSS) − 6.42
	F(2) = 0.65 * Z(BEA) − 0.23 * Z(CEC) − 0.36 * Z(RPE) − 1.19	F(2) = 0.13 * Z(ERSS) − 1.10
	F(3) = 2.41 * Z(BEA) + 3.98 * Z(CEC) + 2.36 * Z(RPE) − 5.93	F(3) = 7.75 * Z(ERSS) − 5.8
(99.6%)		
Senior (98.9%)	F(1) = −3.65 * Z(BEA) − 3.1 * Z(CEC)-2.92 * Z(RPE) − 6.57	F(1) = −8.64 * Z(ERSS) − 6.55
	F(2) = 0.85 * Z(BEA) + 0.26 * Z(CEC) − 0.27 * Z(RPE) − 1.26	F(2) = 0.85 * Z(ERSS) − 1.15
	F(3) = 3.59 * Z(BEA) + 2.82 * Z(CEC) + 2.97 * Z(RPE) − 6.2	F(3) = 8.33 * Z(ERSS) − 6.17
(96.3%)		
All experience levels (99.4%)	F(1) = −3.67 * Z(BEA) − 3.18 * Z(CEC) − 2.36 * Z(RPE) − 6.28	F(1) = −8.31 * Z(ERSS) − 6.27
	F(2) = 0.32 * Z(BEA) + 0.23 * Z(CEC) − 0.28 * Z(RPE) − 1.14	F(2) = 0.29 * Z(ERSS) − 1.11
	F(3) = 3.28 * Z(BEA) + 2.91 * Z(CEC) + 2.89 * Z(RPE) − 6.08	F(3) = 8.12 * Z(ERSS) − 6.04
(98.2%)		

F(1) is the function for the low-level cluster, F(2) is the function for the mid-level cluster, and F(3) is the function for the high-level cluster.

In order to calculate the educational research level of a rural teacher, their original score on the scale is converted to a standardized score in accordance with the table of norms (Table 4), and this standard score is then substituted into the three functions to obtain an output value (Table 6). Teachers are classified according to the maximum value of the function. For example, if a teacher has the highest function output value among the high-level group, his or her level of teaching and research accomplishments is very good.

In addition to the construction of discriminant functions, this type of analysis also produces other interesting findings. First, analysis of the standardized discrimination coefficient and structural coefficient corresponding to each discriminant variable in the first discriminant function indicated that CEC was the strongest discriminating factor. This means that CEC provides the best representation of overall educational research level among rural teachers. Second, the data show that the functions constructed on the basis of both factor scores and total scores were effective (the correct classification rate exceeded 96%). Analysis of the Fisher function based on factor scores showed that the collinearity of the classification coefficients of BEA, CEC, and RPE for each function was very high. This further corroborates the equivalence of the first- and second-order models of educational research achievement.

## 4. General discussion

### 4.1. Structural components and level of educational research among rural teachers

In this study, the structure of rural teachers’ educational research was explored by categorizing the methods used in their educational research, the objects of this research, and the outcomes of this research. The structural components of rural teachers’ educational research activites were explored from the perspectives of research paradigm, object of research, and research outcomes. The results of the initial data analysis ruled out the universal applicability of the academic research paradigm to rural teachers. Rural teachers’ circumstances may not be suitable to enable them to carry out traditional academic research because of various barriers they encounter (Xu, 2021). Therefore, it is more scientifically valid to study educational research achievements among rural teachers from the perspective of action research that they carry out in the course of their educational practice. Our analysis corroborated the suitability of the action research paradigm as a means of exploring the structure of educational research among rural teachers.

More importantly, educational research activities carried out by rural teachers were successfully divided into three structural components according to the object of research. These components were: educational research on basic educational activities (BEA), educational research involving the creation of an educational community (CEC), and educational research involving the refinement and popularization of educational theory (RPE). This proves that educational research among rural teachers consists of three components: teachers’ individualized education practices, with teaching and management as the object [the focus of Kemmis et al. (1982)]; teachers’ educational research in the domain of collective development, taking the form of collaboration and dialog [the focus of Capobianco et al. (2006)]; and education research involving the compilation and promotion of educational experience by teachers [the focus of Elliot (1991)].

This research on the structure of educational research among rural teacher*s* has shown that, although rural teachers face difficulties such as a heavy work burden, limited resources, and a lack of skills, when their educational research achievements are evaluated by linking educational research to their educational practice, rural teachers are not so lacking in educational research accomplishments. They too engage in rich and effective educational research. Of course, the widespread absence of academic research also indicates that there is room for further development of rural teachers’ educational research skills.

Regarding the level of educational research accomplishments among rural teachers, data from Hunan Province in China show that this level is generally good. Average scores across items representing individual types of project ranged between 3 and 4 (where 3 represents general conformance and 4 represents comparative conformance), and the average score on the BEA dimension exceeded 4, representing a relatively high level of accomplishment. Although there is still much room for the development of rural teachers’ educational research level, their current level is much higher than the general impression within the field, provided that they are evaluated *via* a method that is in line with the reality of rural teachers’ circumstances.

### 4.2. Evaluation criteria for rural teachers’ educational research accomplishments

The results of this study highlight the need to weight the criteria used to evaluate educational research achievements among rural teachers toward practical achievements. In China, the current evaluation criteria reflect this weighting. For example, of the 11 indicators extracted in this study from multiple documents used for evaluation and grading, only 1 indicator was based on academic achievement. However, based on the results of this study, there is still extensive room for improvement in the current evaluation criteria.

First, among the three types of research, research on BEA accounts for the largest proportion, and rural teachers’ accomplishments are greatest in this domain. The BEA dimension also includes the teacher’s self-evaluation and their overall evaluation by students, parents, and colleagues, which means that these unofficial evaluations are more closely related to teaching and management skills in daily practice. This is consistent with the finding that the current index-based evaluation system focuses greatly on day-to-day teaching and management. Regarding the clustering of these evaluations and teachers’ teaching and management activities, a possible explanation is that these evaluations largely reflect teachers’ enthusiasm for and attitude toward their work (Jiao, 2004). This is closely related to their self-efficacy and directly impacts their job satisfaction (Wang et al., 2020). The perception of being evaluated positively provides favorable conditions for teachers to actively carry out educational research in their daily teaching and management activities. However, educational research activities falling under the BEA dimension have strong “process” characteristics, which is particularly clear in the fourth item: “I have a comprehensive understanding of students and teach students according to their aptitudes.” At present, the evaluation indicators used in China focus almost exclusively on the “awards” that teachers have won in teaching and management, i.e., the “results” of their work, while ignoring their focus on the “process” of educational research. This insight could prompt education evaluation authorities to incorporate more “process”-based research carried out by teachers in the domains of teaching and management into the evaluation system.

Second, the CEC dimension was identified as the fundamental factor in differentiating rural teachers on the basis of their level of accomplishment in educational research. In other words, CEC is the key to distinguishing rural teachers from one another in this domain. Carrying out educational research in the area of collective educational practice means that individuals within the collective constantly think about and learn from others’ experience, ideas, opinions, and suggestions (Li and Zhao, 2011). They absorb the wisdom of many actors, which has a twofold impact on the development of individuals and the collective. However, in the evaluation indices used to evaluate rural teachers, CEC has not received the attention it deserves. For example, only one of the 11 indicators extracted in this study from multiple documents used in evaluation and grading was related to collective activities. Inadequate incentives and guidance from authorities, in part, have led to several problems: low willingness to cooperate, a weak atmosphere of collaborative construction, single community member structure, and an imperfect system with imperfect mechanisms underlying its construction (Cai et al., 2020). Therefore, education evaluation authorities should provide more incentives and support for teachers to engage in educational research in their collective activities and achieve accomplishments in this area.

Third, although this research shows that academic papers and monographs are not a suitable criterion for the evaluation of educational research accomplishments among rural teachers, this does not mean that rural teachers do not have the ability to compile their experience for presentation *via* these modes of publication. The results show that rural teachers can also promote their practical experience through educational papers, research reports, and experience introduction. It is worth noting that overall RPE scores were the lowest among all three factors. For a considerable length of time, rural teachers have felt that educational theory is not for them. A series of educational dilemmas have caused teachers to ignore RPE. However, experience compiled and presented by rural teachers is more suitable for education and more conducive to the promotion of sustainable development of rural education. It is important for rural teachers to solve practical problems, and the development of such solutions is an important aspect of rural teachers’ professional development (Zhao, 2018). Therefore, adding the refinement and popularization of their educational experience as evaluation criteria for educational research accomplishments will encourage more rural teachers to join research teams that transform practical experience into theory and also promote this experience.

## 5. Limitations and outlook

Because the experts and respondents who participated in this study were mainly from Hunan Province, the representativeness of the data generated *via* the questionnaire is limited. Moreover, because this research focused on exploration of the structure of educational research activites among rural teachers, rather than the current situation with respect to educational research accomplishments among rural teachers, rural teachers’ teaching and research accomplishments were not compared with those of other groups (such as urban teachers, rural teachers in other countries, or rural teachers in previous years).

Based on this study, the following research directions are feasible for future work: (1) research on the factors influencing educational research among rural teachers and paths by which this influence occurs; (2) research on the improvement pathway followed by rural teachers in terms of their educational research skills and accomplishments; (3) comparative research examining rural teachers’ educational research skills and accomplishments in China as compared to other countries.

## 6. Conclusion

First, educational research carried out by rural teachers is more likely to take the form of action research than academic research. Under the paradigm of action research, rural teachers’ educational research can be divided into three components: educational research on basic educational activities; educational research involving the creation of an educational community; and educational research involving the refinement and popularization of educational theory.

Second, the levels of skill and accomplishment in educational research among rural teachers are generally good, especially in relation to educational research in the domain of basic educational activities. Therefore, the educational research accomplishments of rural teachers deserve more recognition and praise.

Third, rural teachers’ levels of educational research skills and accomplishments increase with increasing teaching experience. When evaluating rural teachers’ educational research skills and accomplishments, their career stage should be taken into account.

Fourth, rural teachers can be divided into three classes according to their educational research skills and accomplishments: high-level, medium-level, and low-level. Engagement in educational research activities involving the creation of an educational community plays an important role in this classification.

Fifth, the criteria used to evaluate educational research skills and accomplishments among rural teachers should be reformed accordingly. The “process attribute” of research on BEA, the overall improvement of accomplishments in CEC, and the self-consciousness of rural teachers in relation to RPE should receive more attention.

## Data availability statement

The raw data supporting the conclusions of this article will be made available by the authors, without undue reservation.

## Author contributions

All authors listed have made a substantial, direct, and intellectual contribution to the work and approved it for publication.

## Funding

This study was supported by Hunan Education Department (grant numbers CX20210426) and Office of Social Science Achievement Evaluation Committee of Hunan Province (grant numbers XSP2023JYZ023).

## Conflict of interest

The authors declare that the research was conducted in the absence of any commercial or financial relationships that could be construed as a potential conflict of interest.

## Publisher’s note

All claims expressed in this article are solely those of the authors and do not necessarily represent those of their affiliated organizations, or those of the publisher, the editors and the reviewers. Any product that may be evaluated in this article, or claim that may be made by its manufacturer, is not guaranteed or endorsed by the publisher.

## Footnotes

^1^Compulsory education includes primary school and junior middle school. The teachers who participated in this study were rural teachers at the compulsory education stage.
